# The advancements in organoids: Potential and challenges in researching the esophagus and esophageal squamous cell carcinoma

**DOI:** 10.1016/j.gendis.2025.101680

**Published:** 2025-05-10

**Authors:** Xinxin Li, Zhuo Wang, Yongpan Liu, Jiaying Zhang, Lijia Zhang, Yi Li, Xiaolu An, Yihui Yang, Ruixuan Yu, Meng Zhao, Kuancan Liu

**Affiliations:** a Central Laboratory, Xiang’an Hospital of Xiamen University, School of Medicine, Xiamen University, Xiamen, Fujian 361102, China; b School of Medicine, Xiamen University, Xiamen, Fujian 361102, China; c School of Life Science, Xiamen University, Xiamen, Fujian 361102, China; d Department of Cardio-Thoracic Surgery, The 900th Hospital of the Joint Logistic Support Force, People's Liberation Army, Fuzhou, Fujian 350025, China; e School of Pharmaceutical Sciences, Shenyang Pharmaceutical University, Shenyang, Liaoning 110016, China; f College of Health Caring Industry of Harbin Medical University (Daqing), Daqing, Heilongjiang 163711, China; g Institute for Laboratory Medicine, The 900th Hospital of the Joint Logistic Support Force, People's Liberation Army, Fuzhou, Fujian 350025, China

**Keywords:** Disease modelling, Drug screen, Esophageal squamous cell carcinoma, Esophagus, Organoid

## Abstract

Esophageal squamous cell carcinoma (ESCC) ranks among the top six deadliest malignancies globally, characterized by an alarmingly low five-year survival rate. This aggressive cancer is especially prevalent in Asian countries, where it is strongly influenced by various factors, including dietary habits. Organoids, a novel bioresource that are three-dimensional, miniature organ-like structures derived from stem cells or cancer cells in a laboratory, closely mimics the architecture and function of actual organs, providing an enhanced model for *in vivo* disease representation. These structures hold immense promise for advancing disease modelling, drug testing, personalized medicine, and investigating intricate biological processes. Nevertheless, numerous challenges remain, warranting further investigation. This review offers insights into the superiority of organoids in ESCC disease modelling, especially in drug screening and treatment optimization. The combination of organoids with gene editing will elucidate mechanisms of ESCC, which will be helpful for early molecular marker discovery and immunotherapy, providing potential strategies for therapeutic targets and personalized intervention.

## Introduction

Esophageal squamous cell carcinoma (ESCC), a predominant subtype of esophageal cancer, is distinguished by its high malignancy and dismal prognosis. This aggressive cancer significantly contributes to global cancer-related mortality, particularly in regions such as East Asia and Africa, where its incidence and mortality rates remain alarmingly high. Each year, over 500,000 new cases of esophageal cancer are reported globally, with more than 90% being ESCC.^1^^,^^2^ The etiology of ESCC is multifactorial, involving environmental influences, viral infections, mutations in specific genes, aberrant gene expression, and dysregulated signal transduction pathways.^3^, ^4^, ^5^ The absence of early symptoms often leads to a late-stage diagnosis, complicating treatment and exacerbating the poor prognosis.^6^

Several factors contribute to the high lethality and poor prognosis associated with ESCC. Firstly, the cancer is highly invasive, frequently penetrating adjacent tissues and leading to lymph node involvement and distant metastases.^7^ Secondly, the oesophagus’s complex anatomy and location create significant challenges for surgical resection, often resulting in incomplete tumor removal and high recurrence rates.^8^ Additionally, ESCC exhibits low sensitivity to chemotherapy and radiotherapy, with drug resistance commonly limiting treatment effectiveness. For instance, homeobox C10 (HOXC10) binds to human Erb-b2 receptor tyrosine kinase 3 (ERBB3) and activates the phosphoinositide 3-kinase (PI3K)/protein kinase B (AKT) signaling pathway, which is essential for cell growth and proliferation. This activation promotes the growth of ESCC cells, making them more aggressive and resistant to treatment. Additionally, HOXC10 binds to Ku70, a protein coded by X-ray repair cross complementing 6 (XRCC6) gene and is essential for DNA repair, and accelerates the DNA repair through the non-homologous end-joining (NHEJ) pathway, a pivotal pathway repairs DNA damage caused by radiation and chemotherapy, indicating that elevated HOXC10 levels in patients with ESCC may predict poorer outcomes following radiation or chemotherapy.^9^^,^^10^

Recently, organoid technology has emerged as a promising tool in cancer research, including ESCC,^11^ pancreatic cancer,^12^ and breast cancer.^13^ Organoids are three-dimensional cell culture systems derived from primary tissues, stem cells, or cancer cells, capable of self-renewal and differentiation, thus mimicking the architecture and function of the original organ *in vitro*. To achieve this, cells are embedded in a specific extracellular matrix (typically Matrigel) and cultured in a medium tailored with microenvironmental factors. ESCC organoids are commonly derived from patient biopsy samples or resected tumor tissues, offering a more accurate representation of the genetic and phenotypic heterogeneity of human cancers.^11^ Advances in organoid culture techniques have significantly enhanced the establishment, expansion, and long-term maintenance of these models, enabling the creation of living biobanks of tumor organoids that facilitate high-throughput drug screening and personalized medicine approaches.^14^ ESCC organoid lines, which preserve the genetic and histological characteristics of the original tumors, have been successfully established, allowing for detailed molecular and functional analyses. These organoids have been instrumental in identifying novel therapeutic targets and assessing the efficacy and toxicity of various anti-cancer drugs, thus providing critical insights into drug development and resistance.^15^ Furthermore, organoids have shown potential in modeling the tumor microenvironment (TME), including interactions with stromal and immune cells, thereby deepening our understanding of tumor biology and treatment responses.^16^

## Current strategies for ESCC treatment and causes of poor prognosis

### Current strategies for ESCC treatment

ESCC is characterized by high mortality and extremely low five-year survival rates. Despite the availability of various treatments, outcomes remain unsatisfactory. Surgical treatment of ESCC presents numerous challenges and limitations. Esophagectomy, the primary curative approach for ESCC, is a complex and high-risk procedure often associated with significant postoperative complications, including bleeding, infection, and the commonly observed anastomotic leakage.^17^ These complications can be life-threatening and may necessitate additional surgical interventions, particularly in elderly patients or those with comorbid conditions.^8^^,^^18^ Additionally, if esophagectomy fails, salvage surgery might be required, which carries a higher incidence of complications.^19^ Despite these challenges, advancements in endoscopic technology have introduced new treatment options for patients with early-stage ESCC. Endoscopic mucosal resection is a less invasive procedure, but it is associated with a lower rate of complete tumor removal, which consequently leads to higher recurrence rates.^20^, ^21^, ^22^ To address these issues, endoscopic submucosal dissection has been developed, offering higher resection rates and reduced recurrence risks.^20^ Furthermore, the combination of endoscopic resection with chemotherapy and radiotherapy has emerged as a novel, minimally invasive treatment strategy for patients with ESCC exhibiting submucosal invasion.^23^ This approach has demonstrated both safety and efficacy. These advancements have expanded the spectrum of treatment options and enhanced the management possibilities for ESCC.

Currently, surgery is the main treatment for early esophageal cancer, but most esophageal cancer patients are already in the locally advanced stage when diagnosed. The effect of surgery alone is very limited, and the 5-year survival rate is only 25%.^24^^,^^25^ Neoadjuvant chemoradiotherapy significantly improves the overall survival rate and complete tumor resection rate of patients with esophageal cancer when compared with surgery alone after meta-analysis.^26^ It is effective for patients with ESCC and esophageal adenocarcinoma (EAC), especially for patients with locally advanced disease, and carboplatin combined with paclitaxel is the first choice (level 1 recommendation).^24^ Before implementation, the patient’s overall condition and tolerance need to be carefully evaluated to cope with the possible increased risk of surgery-related complications. Experiments such as NEOCRTEC5010 and CROSS trials have confirmed that neoadjuvant chemoradiotherapy combined with surgery not only improves the survival rate of patients with ESCC but also reduces the risk of local recurrence and distant metastasis, thereby significantly improving the long-term overall survival rate and disease-free survival rate.^10^ Although neoadjuvant or final chemoradiotherapy has become the standard treatment, the prognosis of patients is still not ideal, and the rate of recurrence is still high.^25^^,^^27^^,^^28^ To improve the overall treatment effect and patient quality of life, future studies should focus on determining the optimal neoadjuvant therapy regimen, further reducing treatment toxicity, and evaluating the efficacy of the treatment strategy for esophageal cancer patients with different pathological types and tumor locations in detail.

### Causes of poor prognosis involve the lack of early diagnostic indicators and tumor resistance to radiotherapy and chemotherapy

The primary challenge contributing to the poor prognosis of ESCC is the delayed diagnosis, often due to the nonspecific nature of early-stage symptoms. This lack of early clinical indicators frequently leads to diagnoses at advanced stages, significantly diminishing treatment efficacy and survival prospects. Although endoscopy has been demonstrated as an effective method for detecting early-stage esophageal cancer and reducing mortality, its invasive nature and high costs restrict widespread use.^29^ Consequently, the development of a non-invasive early diagnostic method is of paramount importance. Several specific and sensitive markers have been identified in the progression of ESCC, including p53, NY-ESO-1, MMP-7, Hsp70, PRDX-6, Bmi-1 autoantibodies,^30^ and miRNA.^31^ However, clinical application has been limited by the small number of patient cases. A diagnostic model based on serum miRNA from 566 patients with ESCC and 4965 control patients represents a relatively large-scale research effort in ESCC.^32^ Nevertheless, the sensitivity and specificity of existing tumor markers for early-stage ESCC remain low, and the lack of a substantial number of supporting case samples underscores the critical need for establishing a reliable tumor model and biorepository.

Drug resistance also poses a significant challenge in radiotherapy and chemotherapy, leading to diminished treatment efficacy, increased recurrence risk, and reduced survival rates. Understanding the molecular mechanisms underlying drug resistance is essential for developing new therapeutic strategies, such as inhibiting resistance-associated proteins, disrupting signal transduction pathways, or designing novel drugs. Chemotherapy resistance in ESCC is a multifaceted issue involving numerous molecular mechanisms and signaling pathways. For instance, reduced expression of the etoposide-induced protein 2.4 (EI24) gene in ESCC tissues correlates with poor prognosis, while the FAT atypical cadherin 1 (FAT1) gene’s expression level is closely linked to the properties of ESCC stem cells and their sensitivity to cisplatin.^33^^,^^34^ Additionally, dynamic methylation of the solute carrier family 7 member 8 (SLC7A8) gene promoter has been identified as a potential therapeutic target for overcoming multidrug resistance.^35^ The overexpression of the cytoskeleton-associated protein 2-like (CKAP2L) gene in ESCC tissues is associated with tumor progression and heightened drug resistance.^36^ Beyond gene expression levels, signal transduction pathways also play a critical role in ESCC drug resistance. The up-regulation of survival pathways such as nuclear factor-kappa B (NF-kB) and PI3K/AKT/mechanistic target of rapamycin (mTOR) in cancer cells enhances cell survival and resistance to treatment.^37^ Moreover, tumor-associated macrophages secrete chemokine C–C motif chemokine ligand 22 (CCL22), which induces cisplatin resistance in ESCC cells by modulating the diacylglycerol kinase alpha (DGKα)/NADPH oxidase 4 (NOX4) axis.^38^ Epithelial-mesenchymal transition further exacerbates chemotherapy resistance by making cancer cells more invasive and less responsive to treatment.^37^

**Figure 1:**
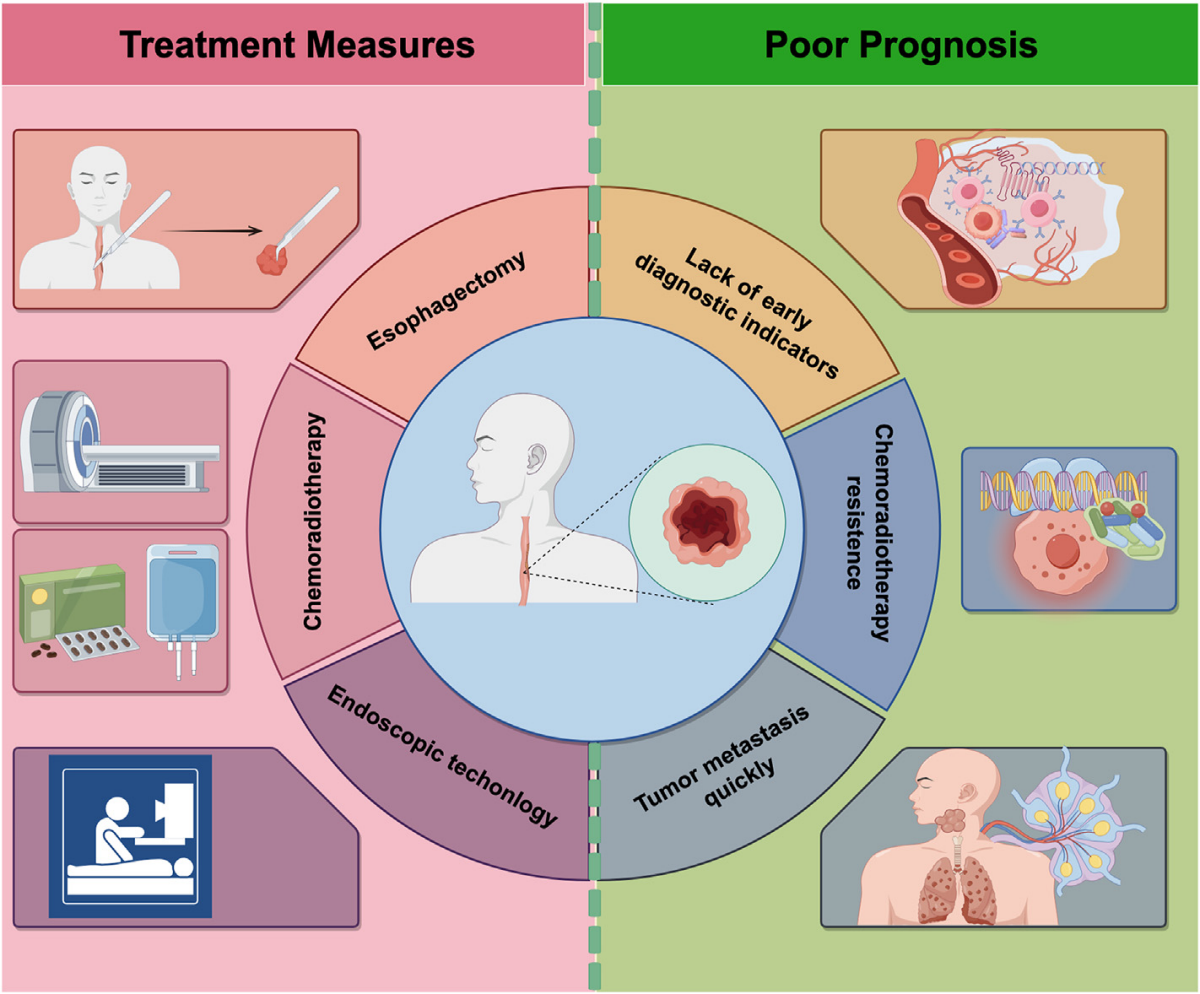
Treatment strategies and primary causes of poor prognosis in Esophageal squamous cell carcinoma (ESCC). The current measures for ESCC treatment are described on the left, and the primary causes of poor prognosis in ESCC are presented on the right.

TME plays a pivotal role in drug resistance in ESCC, and targeting factors within the TME has been suggested as a strategy to overcome resistance.^39^ Key components of the TME, such as fibroblasts, stem cells, and hypoxia, contribute to the complexity of chemotherapy resistance. Recent studies have identified 35 common genes and 4 key genes associated with chemotherapy resistance in ESCC, which are significantly correlated with the half maximal inhibitory concentration (IC50) of paclitaxel, offering potential targets for overcoming resistance.^40^ Thus, chemotherapy resistance in ESCC is a complex process involving multiple molecular mechanisms and pathways, and exploring these mechanisms is essential for identifying new therapeutic targets and strategies to enhance treatment efficacy (Fig. 1).

### Exploring the potential of different models for cancer treatment

To study the mechanism underlying how tumors form and achieve the aim of translation, multiple models, including two-dimensional (2D) cell culture, three-dimensional (3D) cell spheroid culture, *ex vivo* tissue slice culture, tissue engineering and microfluidic chip, and organoid culture, have been established for cancer treatment, they were also used for ESCC, and their advantages and disadvantages among these models are compared and summarized (Table 1).

**Table 1:** Comparison of advantages and disadvantages among different tumour models.

Culture Technique	Advantages	Disadvantages
2D cell culture	Low cost, simple operation, and easy observation	Lacks three-dimensional structure, offers limited physiological relevance, and faces challenges with high cell density
3D cell spheroid culture	Mimics 3D structures and promotes cell differentiation, physiological microenvironment, and tumour study	Requires technical expertise, faces challenges in scalability, and presents concerns with oxygen and nutrient supply
Tissue engineering & microfluidic chip	Mimics organ functions, promotes tissue maturation, and enables accurate physiological simulation and real-time monitoring	Technically demanding, predominantly research-oriented, and associated with high costs
*Ex vivo* tissue slice culture	Effectively maintains the tumour microenvironment and is comparatively easy to manage	Requires specialized expertise, with high variability in sample quality
Organoids	Mimics tissue functions and retains genetic and functional properties, suitable for disease and drug research and personalized cancer treatment	Challenging to scale, involves high material and labour costs, and necessitates specific culture conditions.

### 2D cell culture

In disease modelling, the primary objective is to recreate and elucidate the pathophysiological mechanisms of diseases *in vitro*. While 2D cell culture is a straightforward and viable approach, it is inherently limited by its artificial setting, which lacks the extracellular matrix, thereby hindering the replication of complex 3D intercellular interactions and the multifaceted nature of the TME.^41^^,^^42^ Comparative studies of human skin cell reactivity under oxidative stress and exposure to potentially toxic heavy metals in 2D versus 3D conditions have revealed that cells in 3D culture exhibit significantly enhanced resistance to these harmful factors compared with their 2D counterparts.^43^ Moreover, the limitations of 2D cell culture models in assessing the toxic effects of silver nanoparticles have led to inconsistent results between *in vitro* and *in vivo* assays, indicating that 2D cultures may not accurately reflect physiological cellular responses *in vivo*.^44^ Another study demonstrated the heterogeneity of ESCC samples at the gene mutation and expression levels. By constructing a non-silent protein-coding gene mutation matrix and applying a matching difference function, significant differences in mutation spectra between ESCC cell lines and patient tissues were revealed, and the heterogeneity among patient tissues was more prominent among these differences. High consistency in gene expression was found in ESCC cell lines after transcriptome analysis, while patient tissues exhibited greater discreteness. Therefore, significant genetic heterogeneity among ESCC samples was confirmed, and it was difficult for ESCC cell lines to fully represent the complex heterogeneity characteristics of patient tissues at the mutation and expression levels.^45^

Further emphasizing the limitations of 2D systems, glioblastoma cells cultured within a 3D matrix demonstrated a heightened tolerance to temozolomide compared with those grown in 2D, with this resistance further intensified under hypoxic conditions.^46^ Additionally, differences between 2D and 3D culture models have been observed in the protective effects of phytocannabinoid cannabidiol against UVA/B-induced damage, enhancing our understanding and application of cannabidiol as a UVA/B protectant.^47^ These discrepancies between 2D and 3D cultures raise legitimate concerns about the utility of 2D systems for achieving the fidelity necessary for precise disease simulation.

### 3D cell spheroid culture

The 3D cell sphere model closely simulates key characteristics of human solid tumors, including their structural organization, layered cell assembly, hypoxia, and nutrient gradients. In this model, tumor cells cultured in a suspension medium or matrix gel can spontaneously aggregate to form a spherical structure.^48^ Compared with 2D cell culture, the 3D spheroid culture method more accurately mimics the *in vivo* growth behavior and drug responses of tumor cells. For instance, previous studies have shown that vascular endothelial growth factor (VEGF) mRNA levels, mediated by hypoxia inducible factor 1 subunit alpha (HIF-1α) up-regulation, are twice as high in three-dimensional spheroidal KYSE-70 ESCC cells as in monolayer-cultured cells.^49^ Additionally, the application of 3D spheroid culture technology to the breast cancer cell line MCF-7 has highlighted the critical roles of estrogen sulfotransferase and steroid sulfatase in spheroid formation.^50^ Despite their ability to replicate certain characteristics of human solid tumors, such as drug resistance, three-dimensional cell spheres are limited by their simplified tissue structure, lack of tissue diversity, and the genetic homogeneity of tumor cells. These limitations hinder their ability to fully reproduce the heterogeneity and complex microenvironment of tissues *in vivo*, making them less suitable for modeling complex diseases and conducting in-depth mechanistic studies.

### *Ex vivo* tissue slice culture

*Ex vivo* tissue slice culture is a novel method designed to preserve the original TME and tissue architecture by slicing fresh tumor tissue and maintaining it *in vitro*.^51^ This technique closely approximates *in vivo* conditions, retaining all cell types, including immune cells, and thereby allowing for a more accurate simulation of biological processes. However, the method is technically demanding, requiring precise control over section thickness and culture conditions to maintain cell viability and function. The preparation and maintenance of tissue sections necessitate high technical expertise.^52^ Additionally, the heterogeneity of tissue samples, influenced by variations in tissue source and sectioning procedures, can significantly affect the reproducibility and standardization of experimental results.

For instance, different concentrations of oxaliplatin, cetuximab, and pembrolizumab were applied to liver metastasis sections from patients with colorectal cancer. The findings indicated that all but one patient exhibited dose-dependent sensitivity to oxaliplatin, while only two patients responded to cetuximab and pembrolizumab, respectively.^53^ Similarly, an *in vitro* human adipose tissue slice culture (HATSC) model was developed to study adipose tissue and mechanisms related to obesity. This model includes mature adipocytes, mesenchymal stem cells, stromal tissue, and immune cells. The survival rate of HATSC was confirmed through routine histology, immunofluorescence techniques, functional analysis, and *in vivo* imaging, specifically using Calcein-AM for live cells and propidium iodide for apoptosis/necrosis.^54^ Despite the significant value of tissue slice culture in simulating the TME and advancing the study of tumor biology, its complexity and inherent sample heterogeneity present challenges to the reproducibility and standardization of experimental outcomes. These limitations must be carefully considered in experimental design and data analysis to ensure the reliability and consistency of research findings.

### Tissue engineering and microfluidic chip

Microfluidic chip technology utilizes micromanufacturing techniques to construct chip systems that mimic human organ functions on a microscale.^55^ In this system, tissues are cultured on scaffolds and undergo maturation in bioreactors or under specific culture conditions until their function and structure closely resemble those of natural tissue.^56^ This approach enables the precise simulation of tissue and organ physiological functions within a controlled microenvironment. By manipulating hydrodynamic conditions—such as flow rate and pressure—within the microfluidic system, it accurately replicates blood flow and nutrient exchange and can also integrate sensors for real-time monitoring of cellular physiological states and responses.^57^^,^^58^ A bionic esophageal cancer model was established with hollow microfibers prepared by microfluidic spinning. The adjustability of model parameters and the easy adjustment of mechanical strength, wall thickness, cell density, and degree of infection enable it to accurately simulate the dynamic and reactive physiological ecology *in vivo*. Based on this model, it was convenient to study whether *Porphyromonas gingivalis* promotes the malignant progression of ESCC.^59^

The application of microfluidic chips to simulate tumor growth environments has been explored in various studies, including research on breast cancer.^60^ This technology allows for meticulous control over microenvironmental parameters, resulting in a more realistic tumor model. However, despite its significant potential, microfluidic chip technology remains technically challenging and is currently in the research phase, with limited application in large-scale preclinical studies.

### Organoid culture

**Figure 2:**
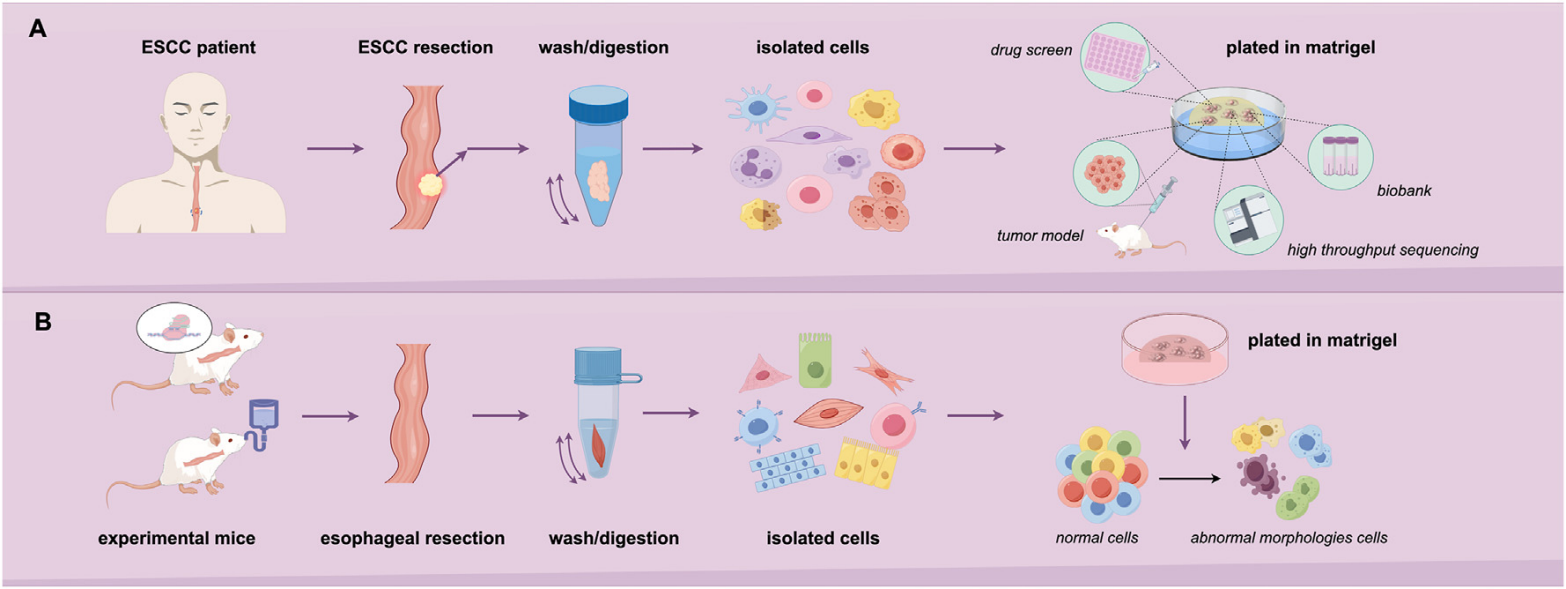
Schematic diagram for isolating and culturing esophageal organoids from mice and humans. **(A)** The protocol of isolating and culturing esophageal organoids from human and their applications. **(B)** The protocol of isolating and culturing esophageal organoids from mice.

Organoids, a 3D cell culture technology derived from primary or stem cells, effectively replicate the functions of tissues and organs as well as the interactions between cells *in vitro*.^61^ Since mouse intestinal adult stem cells were used to culture organoids in 2009, organoid technology has made significant progress.^62^ In 2022, the US FDA approved the first clinical trial of a therapy based on organoid chip research data.^63^ Combined with gene editing and chips, organoids have broken through the limitations of traditional models and brought unlimited possibilities to the medical field. However, studies on esophageal cancer organoids are still limited, with little clinical evidence and a lack of comprehensive investigation on its tissue characteristics. Fortunately, a series of EAC organoids was recently developed to recapitulate tumor characteristics, suggesting that EAC organoid is a powerful tool for studying clonal evolution and provides a valuable preclinical platform for developing precision therapies.^64^ Similarly, the successful establishment of patient-derived organoids (PDOs) in ESCC highlights their value in studying tumor heterogeneity, drug response assays, and mechanisms of drug resistance.^11^^,^^65^ The process of isolating and culturing organoids involves multiple intricate steps. ESCC cells or normal esophageal epithelial cells are firstly isolated from human or mouse esophageal tissues. These tissues are then finely minced and digested using 0.05 % trypsin–EDTA to dissociate the cells, resulting in a single-cell suspension. This suspension is subsequently mixed with Matrigel and cultured in a specialized medium, such as esophageal minimum essential organoid culture medium (Fig. 2). The organoid culture medium is formulated with various components to replicate the microenvironment of ESCC. However, the composition of the medium may need to be tailored depending on the type of organoid and the specific objectives of the study (Table 2). When esophageal organoids derived from KP mice (which carry both potential for Kras^G12D^ expression and Trp53 gene mutations) are infected with Ad-Cre (a type of adenovirus expressing the Cre recombinase enzyme), the knockout of these genes is observed at the cellular level.^66^ The infected organoids exhibit abnormal morphologies and tumor-like characteristics akin to ESCC, including loss of intraluminal keratinization, non-spherical growth patterns, and accelerated proliferation rates (Fig. 2). This esophageal organoid model effectively recapitulates the early lesions of ESCC, offering a crucial experimental system for studying the disease.

**Table 2:** Differences in media between mouse and human oesophageal organoids.

Components	Human ESCC	Mouse oesophageal organoids	Function
DMEM/F12	Base medium^+^	Base medium^∼^	Provides essential nutrients and environment
EGF	50 ng/mL^+^	50 ng/ml^∼^	Promotes cell proliferation
B27	1X^+^	1X^∼^	Provides vitamins, hormones, and other growth factors
N2	1X^+^	1X^ˆ^	Provides trace elements and hormones
Glutamax	1X^+^	1X^∼^	Participates in protein and nucleic acid synthesis and supports cell growth and metabolism
Y-27632	10 μM^+^	10 μM^∼^	Inhibits apoptosis
NAC	1 uM^+^	1 mM^ˆ^	Exerts anti-oxidation activity and promotes cell survival and proliferation
HEPES	0.15 mM^+^	0.15 mM^ˆ^	Maintains pH stability and improves cell environment
Penicillin-streptomycin	1 %^+^	1 %^∼^	Prevents bacterial contamination
Wnt/R-Spondin/Noggin	100/500/100 ng/mL∗	100/100/100 ng/mL^∼^	Promotes self-renewal and proliferation of stem cells
Gastrin	/	100 uM^ˆ^	Promotes cell growth and glucose metabolism
CaCl_2_	1 mM^#^	/	Provides calcium ions essential for cell signalling, adhesion, cytoskeleton organization, and membrane stability
Fungizone	0.5 ug/mL∗	/	Prevents fungal infections in the culture, ensuring a contamination-free environment for organoid growth
SB202190	/	10 μM^∼^	Regulates cell proliferation, differentiation, and apoptosis
Nicotinamide	/	10 mM^∼^	Supports cellular metabolism and NAD synthesis, maintaining cell viability
A83-01	/	500 nM^∼^	Regulates cell proliferation, differentiation, and epithelial–mesenchymal transition

**Human ESCC:** #: reference 81; ∗: reference 101; +: Both.

**Mouse oesophageal organoids:** !: reference 66; ˆ: reference 132; ∼: Both.

Due to their ease of extraction, cultivation, long-term passaging, and cryopreservation, organoids are extensively utilized in tumor treatment research and in-depth mechanistic studies.^67^ Their histological and genetic resemblance to the original tumors, combined with their retention of gene expression profiles and functional properties, enables them to simulate physiological and pathological processes with high accuracy *in vivo*.^68^^,^^69^ Personalized organoid models created from patient-derived cells are particularly valuable for disease research and drug testing, making them highly suitable for personalized cancer treatment. Organoid models have been developed using biopsy tissues from patients with ESCC before and after neoadjuvant chemotherapy, demonstrating significant consistency with the original ESCC tissues in terms of proliferation and drug sensitivity.^70^

Moreover, organoid-based studies have explored tumor immune responses by establishing oral and esophageal organoids from both mice and humans. For instance, an *in vitro* model was developed by co-culturing patient tumor-derived organoids with autologous CD8^+^ T lymphocytes to assess the tumor-killing effects mediated by these immune cells.^71^ The stringent requirements for organoid culture conditions, including the substantial need for matrix gel and other resources, pose challenges to large-scale production. Nevertheless, advancements in micro-engineered cell culture technology have enabled the suspension culture and real-time analysis of numerous independent gastrointestinal organoids, thereby enhancing the consistency and scalability of organoid cultures.^72^ Additionally, the development and optimization of multiple hydrogels as viable alternatives have the potential to reduce costs and streamline the production process.^73^

### The potential of organoids in studying drug response mechanisms, drug screening, and treatment optimization

Tumor cells can evade immune surveillance through various mechanisms, with their distinctive metabolic pathways and the heightened expression of ion pumps on the cell membrane potentially diminishing the efficacy of drugs.^74^, ^75^, ^76^, ^77^ Additionally, tumor heterogeneity complicates treatment, as genetic mutations within a patient’s tumors can result in significant variability, even within the same individual. This heterogeneity leads to inconsistent drug responses across different tumor cells. These challenges underscore the urgent need for new drug development, making the creation of a highly reproducible model that accurately simulates *in vivo* tumor behavior crucial. Organoids have emerged as a promising solution, with PDOs offering the potential to precisely predict tumor responses to drugs in the context of personalized medicine.^78^

### Organoids exhibit genomic stability and preservation of the mutational spectrum

The culture environment of organoids has been significantly optimized compared with other cancer models. Organoids can effectively mimic the differentiation and self-renewal processes of stem cells *in vivo*, minimizing the mutations often seen in long-term cell lines. The use of specific growth factors and small molecule reagents to sustain growth further reduces the stress response or mutation risk associated with *in vitro* culture.^67^ Pathological analysis via hematoxylin-eosin staining of patient-derived breast cancer organoids has shown that the phenotype of these organoids closely mirrors that of the original tumors. Subsequent genome sequencing has revealed that breast cancer organoids possess more abundant DNA copy numbers and clearer genomic signals. These organoids accurately reproduce the copy number variation patterns of the original tumors, with oncogenes generally exhibiting highly correlated signal amplitudes.^79^ The challenges of cancer treatment are largely tied to the high mutation rates in tumor cells, compromised DNA repair mechanisms, genomic instability (including chromosomal structural and numerical variations), and exposure to endogenous or exogenous carcinogens.

Organoids, cultured from cells directly isolated from patients' tissues, retain the mutant spectrum of the original tumor and include various cell types that contribute to the tumor’s heterogeneity. For instance, a comparison of ESCC cell lines with primary ESCC-derived organoids revealed similar growth patterns and morphological characteristics.^80^ Additionally, flow cytometry was employed to assess intra-tumor heterogeneity, demonstrating that cell surface markers and autophagy activity in ESCC organoids and xenografts were highly consistent. This indicates that the culture conditions of 3D organoids can preserve the functional heterogeneity of tumor cells and maintain the mutant spectrum of tumors *in vivo*. Clinically annotated EAC organoid cultures were also generated to recapitulate the morphological, genomic, and transcriptomic features of the primary tumor, including point mutations, copy number variations, and mutational signatures. Organoid cultures have been shown to be polyclonal after karyotyping; they reflect the clonal structure of the primary tumor and provide a preclinical tool for studying clonal evolution and precision therapy.^64^

Cancer cells in ESCC tumors have a high degree of heterogeneity, and cancer cells with high expression of CD44 have stronger autophagic activity, which is associated with poor clinical outcomes in patients. CD44 expression in the tumor was wide-ranging, and CD44 cells with high CD44 expression contained more autophagic vesicles, indicating that they may respond to stress in the TME by enhancing autophagy. In addition, 3D organoid culture conditions may recapitulate the cellular heterogeneity, as 3D organoids and xenograft tumors grown under these culture conditions are similar in CD44 expression and autophagic vesicle content.^11^ These attributes make organoids a valuable tool to provide more accurate and reliable insights into tumor biology, thereby advancing cancer research. Given their ability to replicate the key genetic information and functional characteristics of original tumors *in vivo*, 3D organoids are instrumental in studying mechanisms of drug resistance and optimizing treatment strategies.^66^^,^^81^^,^^82^ Consequently, organoids can largely reflect the response of clinical cancer patients to drugs. Organoid technology holds significant promise for broad applications in cancer research and therapy.

### Organoids have advantages for elucidating the mechanism of resistance to chemotherapy drugs in patients with cancers

Chemotherapy remains a cornerstone in cancer treatment, but drug resistance poses a significant challenge. Drug resistance, which can be categorized into primary and acquired types, refers to the ability of tumor cells to withstand the effects of anti-cancer drugs.^83^ To address resistance to single-drug chemotherapy, multi-drug combination therapy utilizing drugs with different mechanisms of action is commonly employed in clinical settings. While this approach mitigates some clinical challenges, it is still inadequate for managing many tumor types, and there remains a high risk of relapse in patient post-treatment.^84^ Consequently, the development of novel, broadly applicable treatment strategies is of paramount importance. In this context, in-depth studies of resistance mechanisms using organoid technology are essential for advancing more effective therapies. PDO-based drug sensitivity testing can provide important insights into the efficacy of treatment for patients with ESCC. PDOs were successfully generated from a heterogeneous group of ESCC patients, and the drug responses of PDOs were closely correlated with individual clinical outcomes and drug sensitivity profiles, the paclitaxel plus cisplatin-sensitive group had significantly longer progression-free survival compared with the resistant groups, highlighting the potential of PDOs to address clinical translational questions and provide insights into personalized treatment strategies.^65^

### The combination of organoids and gene editing technology contributes to analyzing gene mutations, associated signaling pathways, and epigenetic changes

Tumor cells arise from normal cells through genetic mutations, a process that involves alterations in proto-oncogenes and tumor suppressor genes. The mechanisms underlying drug resistance in tumor cells are largely driven by these genetic mutations. For example, the KRas^G12C^ and BCR-ABL T315I mutations have been identified as key contributors to drug resistance in chronic myeloid leukemia^85^ and non-small cell lung cancer through gene sequencing and drug sensitivity testing.^86^^,^^87^ Specifically, the T315I mutation in the BCR-ABL fusion tyrosine kinase alters the ATP-binding site, leading to continuous activation of the BCR-ABL signaling pathway and subsequent drug resistance in chronic myeloid leukemia cells.^88^ Similarly, the KRas^G12C^ mutation activates the downstream mitogen-activated protein kinase (MAPK)/extracellular signal-regulated kinase (ERK) signaling pathway, resulting in enhanced cell proliferation signals and resistance to conventional therapies.^87^^,^^89^ In addition to genetic mutations, drug resistance can also be influenced by changes in epigenetic regulation. For instance, the expression of histone demethylase lysine-specific demethylase 5A (KDM5A) is significantly up-regulated in resistant cells, and the use of demethylase inhibitors or histone deacetylase inhibitors has been shown to alter the epigenetic state of these cells, thereby reducing their resistance.^90^

The recent integration of organoid culture technology with CRISPR/Cas9 gene-editing techniques offers new avenues for activating or inhibiting endogenous coding and non-coding genes.^91^, ^92^, ^93^, ^94^ This combination enables functional validation of single nucleotide polymorphisms within organoid models. By fusing dCas9 to chromatin modification domains, researchers can modulate the chromatin status of distant candidate functional single nucleotide polymorphisms in target genes. For example, fusions of histone demethylase lysine demethylase 1A (KDM1A) (lysine-specific histone demethylase 1/LSD1) and the KRAB domain from histone methyltransferases with dCas9 have been employed for various applications.^95^ Additionally, in human intestinal organoids carrying the F508del mutation responsible for the rapid degradation of the misfolded CFTR (cystic fibrosis transmembrane conductance regulator) channel protein, CRISPR/Cas9 technology has been used to precisely correct the mutation, thereby restoring CFTR channel activity *in vitro*.^96^

The combination of organoid technology with genome editing tools not only allows for the precise labeling of DNA regulatory elements but also enhances our understanding of tumorigenesis, progression, and drug resistance mechanisms. A platform was established after employing CRISPR/Cas9 technology to create genetic mutations in normal esophageal organoids, and these mutated organoids were transplanted into mice, thereby generating a series of ESCC mouse models. Multiple frequently mutated genes, including E1A binding protein P300 (EP300), FAT1/2/4, lysine methyltransferase 2D (KMT2D), notch receptor 2 (NOTCH2), and transforming growth factor beta receptor 2 (TGFBR2), were validated to serve as tumor-suppressor genes in ESCC. Among these genes, the loss of TGFBR2 significantly promotes the occurrence, development, and multi-organ metastasis of ESCC.^97^ The unique ability of organoids to simulate the TME *in vivo* further positions them as a vital tool for studying and overcoming tumor resistance.

### The combination of organoids with drug sensitivity testing and genome sequencing can dissect the changes in drug metabolism and excretion pathways

Drug susceptibility testing and pharmacogenomic sequencing are typically conducted on stable human cancer cell lines. However, these cell lines are often derived from advanced-stage cancers, introducing a selection bias that limits their clinical relevance. Tumor organoids have emerged as a viable alternative to address this issue, with successful applications in colorectal cancer,^98^^,^^99^ gastrointestinal cancer,^78^^,^^100^ and ESCC.^101^ In colorectal and ovarian cancer organoids, genome and RNA sequencing have identified high expression and gene amplification of multi-drug resistance-related genes, such as ATP binding cassette subfamily B member 1 (ABCB1), which are linked to increased activity in drug excretion pathways.^102^

Drug sensitivity tests on organoid models have revealed resistance to specific chemotherapeutic agents. For example, organoids derived from head and neck squamous cell carcinoma were cultured with a success rate of 30.2%, and IC50 values for cisplatin and docetaxel were determined with organoids through drug sensitivity assays and clonogenic survival assays, indicating that organoids can effectively predict *in vivo* drug sensitivity^103^ and validate the activity of drug efflux pumps.^99^^,^^104^ Combining chemotherapy with drug efflux pump inhibitors partially restored the sensitivity of these organoids to chemotherapeutic drugs, underscoring the role of drug efflux pumps in resistance. This approach not only deepens the understanding of drug resistance mechanisms but also provides a valuable reference for developing personalized treatment strategies. Screening multiple drugs in an organoid model enables the identification of the most effective treatment combinations for individual patients, thereby enhancing clinical outcomes.^105^

### The development of precision medicine for ESCC using organoids

Patients with ESCC face a high recurrence rate following surgery or chemotherapy.^70^ The significant heterogeneity of ESCC results in considerable variation in tumor behavior and treatment responses among patients, with recurrent tumors often differing from the original ones. To address this, most clinical chemotherapy drugs focus on mechanisms that induce DNA damage, inhibit DNA replication, or target biological characteristics such as the rapid division of tumor cells.^106^ However, these treatments often lead to drug resistance and can cause toxic side effects on normal cells due to their lack of specificity.^40^

Precision medicine aims to enhance treatment outcomes by developing personalized treatment plans based on the patient’s genetic and molecular characteristics. This approach involves creating more effective targeted drugs by identifying and focusing on specific genetic mutations or molecular pathways. As previously mentioned, organoids retain most of the patient’s original genomic information, including mutations, making them highly relevant to clinical practice and a valuable tool in the development of precision medicine strategies. The sonic hedgehog pathway was activated in EPC2 cells, an esophageal epithelium cell line that was hTERT-immortalized with functionally intact p53 and p16, after the ectopic expression of E6 and E7 genes within human papillomavirus 16 (HPV16). Coordinated expression of E6 and E7 promoted the growth of EPC2 cells and EPC2-derived organoids, and they also promoted multiple processes of ESCC. Moreover, targeting the carcinogenic effect caused by E6 and E7 proteins with vismodegib inhibitor for the hedgehog pathway achieved therapeutic gains in malignant transformation and progression.^5^

### Organoids are reliable predictors of patients' response to drugs

Biobanks have been established using samples from patients with gastrointestinal cancer, allowing for the comparison of drug responses between cultured organoids and the original patients. The overlap in the mutant spectrum between organoids and their parent biopsies was found to be 96 percent.^100^ Additionally, organoids demonstrate a significant advantage in predicting patients' responses to targeted therapies or chemotherapy.^107^ A similar study revealed that the histopathological and genomic characteristics of organoids closely mirror those of the original rectal cancer samples, further highlighting their relevance in clinical applications.^108^

### The potential of organoids in clinical use and development of early molecular markers

Tumor organoid libraries and adjacent non-cancerous tissue libraries can be established to serve as valuable resources for personalized cancer treatment.^109^^,^^110^ As previously discussed, PDOs represent the most accurate *in vitro* models for replicating the tumor state, preserving the genetic characteristics of the original tissue to a significant extent. By performing drug sensitivity tests and high-throughput drug screening on these organoids, it becomes possible to identify the most effective drug or combination of drugs for a specific patient and subsequently determine the optimal and safest drug dosage. This approach minimizes the toxic side effects of treatment on patients. For example, in a study involving nine EAC organoids, 24 anti-cancer drugs were tested, and the dose–response curves were used to evaluate drug efficacy and determine the effective and safe concentration ranges.^64^ Additionally, ESCC organoid culture system was established from patients after optimization, and the therapeutic effect was validated on ESCC organoids caused by polysaccharides derived from *Agaricus blazei murrill* and *Enteromorpha prolifera*.^111^ This method is equally applicable to ESCC; once an ESCC organoid library is established, similar drug testing and screening can be conducted to identify the best treatment strategy.

Throughout the treatment process, an adjacent non-cancerous organoid library can be utilized for comparison with tumor organoids to elucidate changes in cancer-related gene expression, cell behaviors in specific patients, and differences in drug treatment outcomes. This comparison aids in identifying early molecular markers of cancer and in developing new diagnostic tools.^112^ Additionally, patient samples can be periodically collected during treatment for organoid culture, allowing for the monitoring of dynamic treatment responses. This enables clinicians to maintain flexibility in their approach, making timely adjustments to treatment plans and addressing issues such as disease progression and the emergence of drug resistance.

### The application of organoids in immunotherapy

Organoid models have emerged as a promising tool for investigating the optimal combination of targeted therapy and immunotherapy, which holds significant potential for enhancing treatment outcomes and advancing future research. Recently, a method was developed to generate tumor-reactive T cells for colorectal cancer and non-small cell lung cancer by co-culturing organoids with peripheral blood lymphocytes. These T cells not only respond to cancer in a personalized manner but also establish a platform for analyzing cancer specificity, positioning organoids as a novel model and research platform for tumor immunotherapy.^113^ This platform facilitates the isolation of tumor-reactive T cells and the assessment of cancer cell susceptibility to T cell attacks. Furthermore, this method applies to ESCC research, where organoid models can be employed to gain a deeper understanding of the mechanisms underlying responses to targeted therapies and immunotherapies. This approach provides a foundation for developing individualized treatment plans, thereby improving therapeutic outcomes for patients with ESCC and advancing scientific research in related fields. In addition, a novel air-liquid interface patient-derived tumor organoid culture system has been successfully developed, which could replicate the complex architecture of tumors, including the extracellular matrix and immune compartments, in multiple tumor types.^114^, ^115^, ^116^ T-cell receptors have been shown to be highly conserved between patient-derived tumor organoid cultures and the original tumors, and patient-derived tumor organoid cultures can functionally recapitulate programmed cell death protein 1 (PD-1)/programmed cell death ligand 1 (PD-L1)-dependent immune checkpoints.^113^ This system offers a valuable model for studying the interplay between immune cells and tumors, further contributing to the development of effective cancer therapies.

### The superiority of organoids in studying cell–environment interactions

Organoids closely replicate the *in vivo* TME, including the complex interactions among blood vessels, immune cells, and stromal cells.^16^^,^^117^ Intercellular communication is fundamental to cellular processes such as proliferation, migration, and homeostasis. By adjusting the culture conditions and environment of organoids to promote angiogenesis or modulate inflammatory factor activity, it is possible to enhance drug efficacy. Although there is a substantial understanding of organ microstructure and matrix composition, there is an urgent need to develop complex models that can accurately study heterocellular interactions in cancer research. These models are crucial for investigating the TME, tumor spread, and the key molecular pathways and causal relationships involved in carcinogenesis. ESCC, known for its heterogeneity and unique TME, requires special attention to the intricate interactions among tumor cells, fibroblasts, immune cells, and vascular endothelial cells. These interactions not only drive tumor progression but also contribute to drug resistance through complex signaling networks. To effectively evaluate intercellular communications in ESCC, it is essential that the ESCC organoid model accurately reproduces cellular heterogeneity within a diverse microenvironment.

The ideal 3D culture model should mimic the tissue-specific physiological environment, supporting cell proliferation, aggregation, differentiation, and interactions both among cells and between cells and the extracellular matrix.^118^ While traditional organoid culture methods have limitations in replicating the multicellular characteristics of the TME, these shortcomings have been partially addressed by innovative approaches. For example, PDO cultures using the air-liquid interface can retain immune cells and facilitate co-culture with patient-derived immune cells or cancer-associated fibroblasts, thereby better simulating the complexity of the TME.^16^

## Challenges of organoid models in future cancer research and treatment

**Figure 3:**
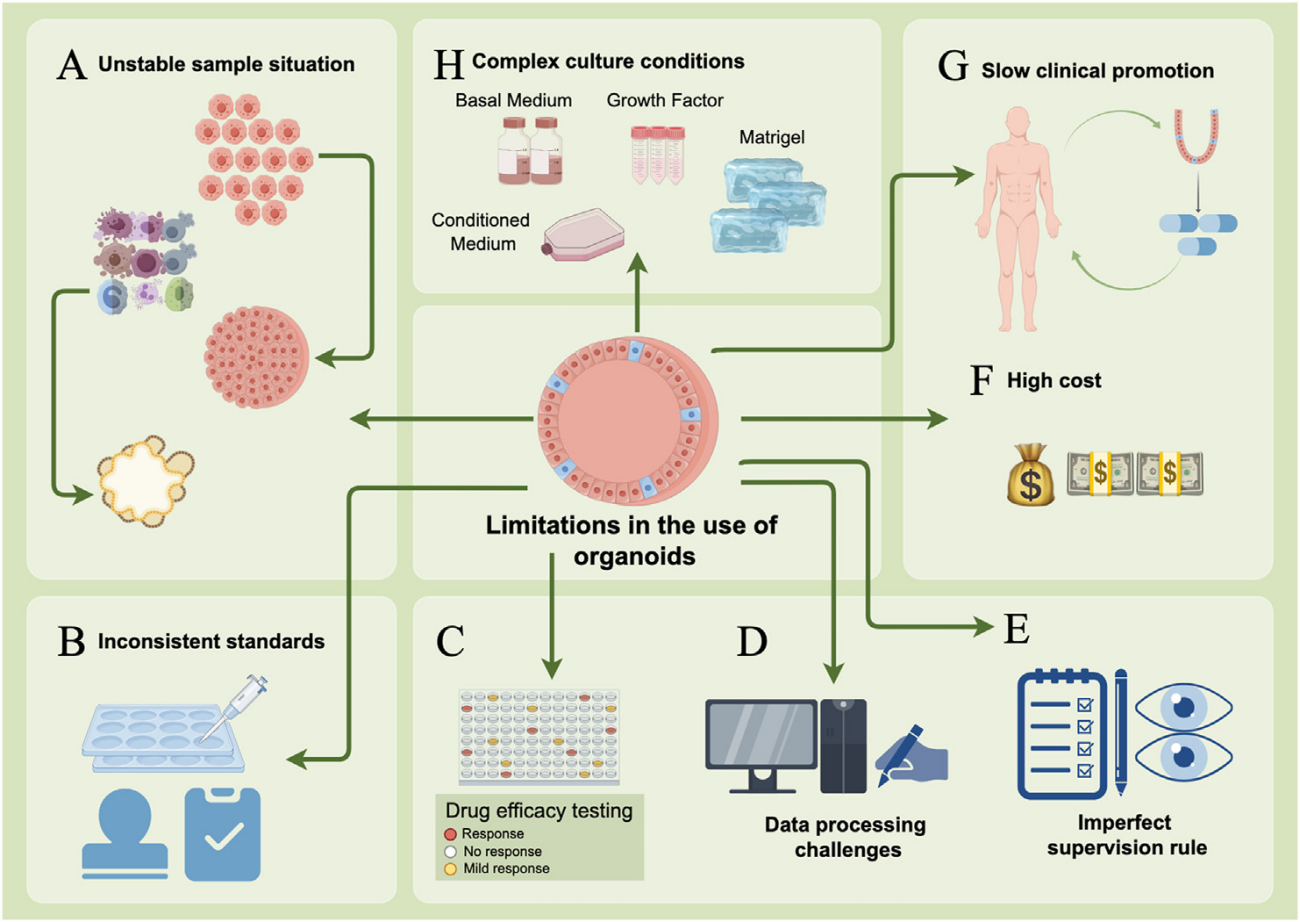
Multiple limitations in the application of organoids. The limitations in the application of organoids include unstable sample situations, inconsistent standards, differences in drug efficacy testing, data processing challenges, imperfect supervision rules, high cost, slow clinical promotion, and complex culture conditions.

The ability of PDOs to accurately mimic therapeutic responses in patients positions these models as valuable tools throughout the pharmaceutical development process and in clinical practice. However, several challenges must be addressed to fully integrate organoid model systems into clinical settings. These challenges include accelerating organoid development, enhancing success rates in organoid establishment, reducing associated costs, increasing experimental yield, and improving reproducibility (Fig. 3). Overcoming these obstacles is essential for the effective transition of organoid technology from research to routine clinical application.

### Complexities in organoid data integration and analysis

Currently, the establishment of standardized organoid data repositories remains an unmet need. Human and murine organoid models, derived from both healthy tissues and non-cancerous diseases, have yet to be consolidated into a single comprehensive database. The recent launch of the Human Cell Atlas-Organoid initiative, which aims to document all human-derived organoids, marks significant progress in this area.^119^ However, organoid research data present complexities due to the diversity of sources and formats, inconsistencies in technical platforms and standards across laboratories, and variability in data quality arising from different experimental designs. Moreover, the vast amount of data generated by organoid studies, such as high-throughput sequencing and drug screening results, demands advanced computing power, robust storage solutions, and specialized data processing tools.^120^ Currently, there are limited tools and platforms specifically designed for the integration of organoid data, making the interpretation and application of these datasets a complex challenge. Addressing these issues will be essential for advancing organoid research and its application in clinical and pharmaceutical contexts.

### Non-uniform standards and poor repeatability in organoid culture

The cultivation of various organoid types necessitates specialized culture conditions, with different laboratories employing unique combinations of media, culture methods, and growth factors. This variability impacts organoid functionalities and compromises the uniformity of experimental results. Consequently, optimizing and standardizing these cultural conditions is a significant challenge. Advancing the standardization and automation of organoid studies, particularly in the areas of culture conditions and technologies for uniform organoid generation, is essential for enhancing reproducibility and establishing a solid foundation for the commercial popularization of PDO technology.

The difficulty in comparing findings across different studies is exacerbated by the individualized optimization of growth factor concentrations and disparities in microenvironmental conditions.^121^ Additionally, the development of software capable of quantitatively analyzing experimental data is vital for integrating PDOs into clinical decision-making processes, ensuring that organoid-based research can be effectively translated into practical applications in patient care.^122^

### Lack of tissue complexity and precision in organoids

Although organoids offer a more precise representation of *in vivo* tumors compared with traditional cell lines, they remain limited to epithelial cells and lack the diverse cell types present in the TME. This absence means that organoids do not fully capture the complexity of human organs. The TME, which includes components such as the vascular system, neural network, and immune system, plays a critical role in influencing drug responses, either positively or negatively. This limitation in organoid models helps explain the discrepancies often observed between *in vitro* drug sensitivity and *in vivo* outcomes.^123^ For instance, the lack of essential elements like blood vessels, neural networks, and immune cells in organoids raises questions about whether they can truly represent the diversity and complexity of tumor cells *in vivo*. This gap highlights an important area of exploration: the need to more accurately replicate the TME within organoid models. Future research should focus on incorporating processes such as angiogenesis and inflammatory factor infiltration into organoids. Addressing these challenges will enhance the biological relevance and practical applications of organoid models, making them more effective tools for studying tumors and developing treatments.

### High cost in organoid culture

The financial aspect of organoid culture is a critical consideration for the clinical application of PDOs. The high costs associated with organoid culture primarily stem from the time-consuming and labor-intensive processes required for establishing and subculturing PDOs, as well as the expense of essential growth components. These components include costly growth factor combinations and animal-derived substrates such as Matrigel. To reduce these expenses, the development of synthetic matrices has been proposed, with polyethylene glycol hydrogels emerging as a potential alternative to Matrigel.^124^^,^^125^ However, Matrigel currently outperforms all synthetic substitutes in terms of supporting organoid growth. Despite this, the adoption of synthetic scaffolds is expected to lead to more economical and consistent outcomes in the future. Additionally, the integration of microfluidic technology offers a promising breakthrough.^126^ By combining these advanced techniques, the scale of organoid assays can be significantly reduced, enabling rapid screening within a shortened timeframe of just one to ten days. This approach not only holds the potential to lower costs but also to accelerate the pace of research and clinical applications of organoid models.

### Challenges of PDO application in studying ESCC

With the development of organoid culture technology, the success rate of establishing gastric cancer organoids is as high as over 90%, significantly exceeding the 31.25%-to-70% range of esophageal cancer.^127^ The difference reveals the uniqueness in the process of organoid culture according to different cancer types. Given the complex biological characteristics and high heterogeneity of ESCC, it is urgent to optimize the culture environment of tumor and healthy organoids. Although the organoid model can well reproduce tissue structure, it also has shortcomings in microenvironment remodeling, especially the lack of immune cells. Air-liquid interface system-cultured organoids can retain immune cells in the short term, while their limitations on long-term preservation have seriously affected the continued investigation of the ESCC microenvironment.^16^^,^^115^^,^^116^ Therefore, future studies should focus on the optimization of the co-culture system to achieve a more comprehensive simulation of the patient’s microenvironment.

In addition, given the resistance of ESCC to traditional treatments, the potential application of organoids in immune checkpoint inhibitor testing and cell therapy is particularly critical, but the study in this field is still lacking. Although many tumor organoids have been successfully established, there is not much data to determine the potential correlation between organoid-derived drug testing results and clinical responses. Therefore, prospective, comprehensive cohort studies in cancer research, especially in ESCC research, should be performed to promote the development of personalized precision medicine and the optimization of ESCC treatment scheme.

## Conclusions and future perspectives

ESCC poses significant challenges in both diagnosis and treatment due to its asymptomatic nature at early stages, often leading to delayed detection, poor treatment outcomes, and low survival rates. Surgical resection remains the gold standard for ESCC treatment; however, the difficulty in early detection often results in diagnosis at a late stage, thereby increasing the risks associated with surgery and postoperative complications. While endoscopy is an effective method for the early detection of esophageal cancer, its invasiveness and high cost limit its widespread use.^29^ The advent of endoscopic techniques, such as endoscopic mucosal resection and endoscopic submucosal dissection, offers minimally invasive treatment options, but their application is also restricted. Although ongoing research has identified various potential biomarkers for ESCC, their clinical application remains constrained by the small sample sizes used in related studies.

The intrinsic resistance of ESCC cells to radiotherapy and chemotherapy presents a significant challenge, as it diminishes treatment efficacy and heightens the risk of recurrence.^40^ Therefore, understanding the molecular mechanisms underlying drug resistance is critical for developing new treatment strategies, and ongoing research is focused on innovative methods to overcome this resistance. Although tumor models such as 2D and 3D cell spheroids retain some complexities of the TME, they fall short of fully replicating the intricate cell interactions and overall complexity of the *in vivo* TME. Tissue slice culture methods partially preserve the TME *in vitro*, but they face challenges in standardization and methodological requirements. While tissue engineering and microfluidic chip technologies can simulate organ functions, these approaches remain primarily in the research phase due to technical challenges. Organoid culture techniques, which utilize primary cells or stem cells to replicate tissues or organs, offer promising tools for disease research and personalized medicine.^128^ However, these techniques still face challenges in accurately simulating the *in vivo* environment and disease processes.

Previously, a peptide aptamer library and a method for screening specific peptide aptamers were established, and two novel peptides targeting the SRY-box transcription factor 2 (SOX2) protein and the SOX2-CDP (homeobox protein cut-like 1) protein complex were identified.^129^^,^^130^ Additionally, 3-ABA and metformin were also repurposed for ESCC therapy,^131^ while their therapeutic effects on patients with ESCC have not yet been tested. Organoids have emerged as powerful tools for drug screening and treatment optimization, as they effectively simulate the TME and heterogeneity. The use of ESCC organoids derived from clinical samples could be instrumental in verifying the therapeutic efficacy of these drugs in future studies.

Compared with traditional 2D cell cultures or patient-derived xenografts, organoids offer a more accurate representation of genetic mutations and drug responses.^80^^,^^132^ By preserving the mutational spectrum and functional heterogeneity of the original tumor, organoids provide an invaluable model for studying cancer mechanisms and drug resistance. The integration of gene-editing technologies with organoid models enables the exploration of specific mutations and signaling pathways involved in drug resistance.^66^ Furthermore, epigenetic changes, including histone demethylation, which are associated with drug tolerance, can be thoroughly investigated using organoids. Organoids also facilitate the testing of drug sensitivity and the elucidation of changes in drug metabolism and excretion pathways. For instance, organoid models have been employed to study resistance to certain chemotherapy drugs, and targeting drug efflux pumps has been shown to mitigate this resistance.^11^ Thus, organoid models are highly valuable for predicting drug responses, personalizing treatment, and potentially improving treatment outcomes.

Therefore, organoids preserve the genomic information of the original patient and can be used to predict individual responses to drugs. Their close resemblance to the histopathology and genetic characteristics of the original tumor makes them invaluable for testing drug sensitivity and identifying the most effective treatment strategies. Organoids represent a promising tool in cancer research and personalized medicine, providing a reliable platform for dissecting the mechanisms of tumor formation and improving treatment outcomes. They are particularly beneficial in the development of precision medicine for ESCC, a disease marked by high heterogeneity and variability in treatment responses.

Additionally, organoids facilitate the identification of early molecular markers of cancer and the development of new diagnostic tools. They also enable the study of combined targeted and immunotherapies, enhance our understanding of tumor–microenvironment interactions, and simulate the complex TME to boost drug efficacy. Organoids are essential for studying ESCC and establishing personalized treatment plans. However, the future of cancer research and treatment will require addressing challenges related to the integration and analysis of organoid data, the development of standardized and reproducible methods, the management of organoid biological complexity, and the scaling up of organoid production. Overcoming these challenges is essential for enhancing the accuracy, reliability, and clinical applicability of organoid models, thereby advancing our understanding of cancer’s complexity and improving treatment approaches.

## CRediT authorship contribution statement

**Xinxin Li:** Writing – original draft. **Zhuo Wang:** Writing – review & editing. **Yongpan Liu:** Writing – review & editing. **Jiaying Zhang:** Writing – review & editing. **Lijia Zhang:** Writing – review & editing. **Yi Li:** Writing – review & editing. **Xiaolu An:** Visualization, Investigation. **Yihui Yang:** Visualization. **Ruixuan Yu:** Visualization. **Meng Zhao:** Conceptualization. **Kuancan Liu:** Funding acquisition, Conceptualization.

## Funding

This work was supported by the 10.13039/501100001809National Natural Science Foundation of China (No. 82273044), the 10.13039/501100005270Science Fund for Distinguished Young Scholars of Fujian Province, China (No. 2021D034), the Science Fund from the Health Commission of Fujian, China (No. 2023GGB04), and the Clinical Investigation Program from the 900 Hospital (China) (No. 2020L09).

## Conflict of interests

The authors declared no conflict of interests.
